# Humiliating

**Published:** 1855-05

**Authors:** 


### HUMILIATING.

Quem Deus vult perdere, prius dementat.

"*The University of Michigan* would seem to be doomed. As one of the first fruits of placing a mediocre at its head, and a disciple of Hahnemann, at that, who dubs himself "Chancellor," in his characteristic arrogance; we now learn that the Legislature of that State have appended a Professorship of Homœopathy! to the faculty of the Medical School! If the other teachers, thus insulted, do not resign forthwith, their personal status in the profession, as well as that of their school, will speedily be defined. We congratulate Dr. Allen on his escape, for a 'private station is surely the post of honor, when evil men bear rule.' Clairvoyance and Spirit Rapping should next be inaugurated."

The above we find in the "American Medical Gazette." We concur with the editor in the opinion that such acts are humiliating to the profession, and not humiliating *only*, but outrageous and monstrous.

This comes of the government by non-medical men of the department of instruction in medical institutions. In its financial or civil condition, it may be well enough, but the fitness of men, as medical teachers and instructors, can be judged of most correctly by medical men themselves.

There are those who are good physicians, with a large amount of practice and possessing much practical tact who do not possess the necessary qualifications for teachers. Besides other necessary requisites, the applicant should possess sound morals, and be endowed with a large share of the principles of honor and integrity. The example of one who, by his conduct contemns these, tends to weaken and corrupt the moral sentiments of young men upon whom they look for instruction in medical science. A loose and careless expression, or a single sneer of contempt for morals and virtue might exert an unhappy influence upon the minds of those whose characters are not yet fixed nor confirmed for good in after life. One single individual in the faculty of a school devoid of principle, of honor, or honesty, is calculated by his acts and his influence to poison its existence and cause it to decline during his connexion with it.

At the present time when our country is overrun with empericism embracing its various aspects, features and phazes, it is not surpassing, although much to be deplored, that advocates for the different systems should be found in legislative halls. Although they may differ in the peculiar faith among themselves, there is one particular in which they do agree, and that is in *an uncompromising hostility to the regular system.* The life-blood of each depends upon the success with which they can create popular prejudice and even indignation against it.

In Michigan, they have established, upon a liberal endowment, a university, and here are taught the various branches requisite to a professional scholarship. Suppose some one member of the legislature would move to instruct the trustees to dispense with some of these branches because of the absurd (to him) fallacy that the earth was a sphere, while, to his comprehension, it was plain, that bating a few irregularities upon its surface, it was as flat as a "*pancake.*" With the same show of propriety and intelligence they could recommend the establishment of a chair of Homœopathy.

With just as much consistency, wisdom, and justice, would the trustees of any theological seminary in the country, create a department for the elucidation and discussion of the sublime truths of Mormonism, Millerism, Fanny Wright-ism, infidelism, or any other *ism*, and place conspicuously in the library the Mormon Bible—the infidel productions of Paine, Voltare, and others.

Would the learned and pious men of such an institution longer remain where such heretic and false dogmas were forced into the curriculum of the studies in a Christian institution? If they were sincere in their faith, they certainly would not, but would fly from it as if a deadly pestilence had entered its halls. Honesty, honor, duty, a decent respect for their professions, and a regard for themselves, would forbid that they should witness the sacrilege; and so also should the Faculty of the Ann Arbor School of Medicine. They cannot consistently or honorably remain, for such a heterogeneous commingling would degrade the profession from its high position and attaint it with dishonor. They should not witness the sacrilege no more than a true Christian would look upon the performance of some of the solemn and sacred ordinances of the Christian church by the hands of one who had cast a foul aspersion upon the Christian faith and belief, in lauding the infidelic writings of Thos. Paine upon the birth-day anniversary of that distinguished champion of materialism and infidelity.

An attempt was made during the last session of the Legislature of *this* State, to procure the passage of a law, which would have resulted in the disorganization of the Medical Department of the State University. In order to success in the accomplishment of this plot, it was covered up under the deceptive and hypocritical garb of friendship for the institution. No honorable member would have been found who would have proposed the plan, unless he believed it was to promote its interests, and consequently it was made in good faith by him to whose hands it was entrusted. He said he was assured that it was for its benefit when asked to urge the proposition. The dark plot was discovered, however, and crushed in its germ by the wisdom and sagacity of those who, knowing well who was at the bottom of it, and for whose benefit it was to enure, suspected the motives, and hence they scouted the proposition.

The design was to deny the Faculty even the poor priveledge of nominating competent men to fill vacancies when they should occur and also to appoint a Board favorable to certain interests. The Legislature believed that the Faculty were more competent to select their associates than non-medical men, and even if they were not, they should not be forced to take among them individuals with whom a decent self-respect would forbid that they seould act and co-operate.

Some such dark spirit, doubtless, was the mischievous agent in the recent disgraceful movement in the Michigan Legislature who, because his want of merit lay in the way of the gratification of his unhallowed and mercenary ambition; who, because his standing would give him no claim to enter the door by the sun-light of day, sought this under-current mode of destroying its usefulness, and its bright prospects in the future. We have learned to loathe such beslimed reptiles, whose malignity and hypocracy is equalled only in intensity by the bright greenness of the grass in which they are concealed, and in which they creep. A darkling envy for the position and prominence of the incumbents of the respective chairs, and a consciousness of inferiority when compared with their virtue, merits and standing, doubtless prompted the base design of destroying that in which their own demerits denied them an honorable participation.

